# A *Drosophila* heart optical coherence microscopy dataset for automatic video segmentation

**DOI:** 10.1038/s41597-023-02802-y

**Published:** 2023-12-09

**Authors:** Matthew Fishman, Abigail Matt, Fei Wang, Elena Gracheva, Jiantao Zhu, Xiangping Ouyang, Andrey Komarov, Yuxuan Wang, Hongwu Liang, Chao Zhou

**Affiliations:** 1 Washington University in St. Louis, Department of Computer Science and Engineering, St. Louis, MO 63130 USA; 2 Washington University in St. Louis, Department of Biomedical Engineering, St. Louis, MO 63130 USA

**Keywords:** Optical imaging, Optogenetics, Machine learning, Image processing, Heart development

## Abstract

The heart of the fruit fly, *Drosophila melanogaster*, is a particularly suitable model for cardiac studies. Optical coherence microscopy (OCM) captures *in vivo* cross-sectional videos of the beating *Drosophila* heart for cardiac function quantification. To analyze those large-size multi-frame OCM recordings, human labelling has been employed, leading to low efficiency and poor reproducibility. Here, we introduce a robust and accurate automated *Drosophila* heart segmentation algorithm, called FlyNet 2.0+, which utilizes a long short-term memory (LSTM) convolutional neural network to leverage time series information in the videos, ensuring consistent, high-quality segmentation. We present a dataset of 213 *Drosophila* heart videos, equivalent to 604,000 cross-sectional images, containing all developmental stages and a wide range of beating patterns, including faster and slower than normal beating, arrhythmic beating, and periods of heart stop to capture these heart dynamics. Each video contains a corresponding ground truth mask. We expect this unique large dataset of the beating *Drosophila* heart *in vivo* will enable new deep learning approaches to efficiently characterize heart function to advance cardiac research.

## Background & Summary

*Drosophila melanogaster* (a fruit fly) is a powerful model organism for investigating biological mechanisms of human diseases. The fully sequenced *D. melanogaster* genome contains functional homologs for about 75% of human disease-causing genes^1,2^. The fruit fly’s short life cycle, ~ 11 days from embryo to adult, makes it a good candidate for studying age- and disease-related physiological changes. The heart, located ~100 μm below the dorsal surface, is easily accessed by optical imaging. Oxygen transport in *Drosophila* is independent of the tubular heart^3^, enabling the creation of severe cardiac defects without causing death. This independence also enables pacing experiments that can completely stop the heartbeat or accelerate the heart rate. In building an innovative research platform for cardiovascular studies, our research group has developed an integrated apparatus that can simultaneously perform optical imaging and optogenetic pacing to control and characterize the fly heartbeat rhythm at all developmental stages^4,5^. The technique employs genetically modified flies and a custom-built optical coherence microscopy (OCM) imaging system.

Optical coherence tomography (OCT) noninvasively provides micron-scale cross-sectional and 3D images of biological tissues in a wide variety of clinical applications, including ophthalmology^6,7^, cardiology^8–10^, endoscopy^11–13^, dermatology^14–16^, and dentistry^17–19^. OCM^20^, which integrates OCT and confocal detection, offers high resolution at an image penetration depth of around 500 µm, deep enough to image the larval, pupal, and adult *Drosophila* heart. For functional analysis, M-mode images are acquired with repeated frames showing cross-sectional views of the heart at a fixed location over time. In our study, each ~32-second recording captures 4000 2D images at a frame rate of ~125 frames per second. This 2D+ time OCM video acquisition is less sensitive to sample motion and supports higher frame rates, enabling more accurate measurements, especially of fast beating hearts.

To quantify the cardiac function, the heart region in the OCM videos must be segmented accurately. The heart mask that stores the segmentation over time can be used to calculate dynamic cardiac parameters, such as the heart rate (HR), end-diastolic diameter (EDD), end-systolic diameter (ESD), and fraction shortening (FS), all of which characterize the heart function. For cardiovascular research involving small organisms such as *Drosophila*, there are several challenges to quantify the heart function: adequate spatiotemporal resolution with minimum invasiveness, and reliable automatic algorithm for batch processing. Previous techniques using a digital camera or electrophysiological recordings are able to resolve the heart function for quantification, but require invasive dissection procedures to expose the heart^21^. Ocorr *et al*. developed a technique for measuring characteristics of heart rhythmicity^22^. These flies must have ventricle cuticle and organs below the dorsally located heart to be removed to create an unobscured recording, and a camera is used to record heart movement from a top-down approach. This algorithm relies on differences in pixel intensity and overall frame brightness to detect changes in heart wall movement. It has been useful in defining the heart rate, systolic and diastolic index, arrhythmicity, and fractional shortening. The heart recordings usually have big data size and repeated measurements from different individuals, which raises a pressing need for generalized computational methods to autonomous processing. Originally, heart beats were counted manually^23^, which was an incredibly time consuming and less precise technique. Semiautomatic optical heartbeat analysis (SOHA) has been developed by Fink *et al*.^22^ and Ocorr *et al*.^21^ and upgraded by Monck *et al*.^24^ to retrieve the fly heart coherent movement. This method has low implementation cost and can well characterize heart beating, but did not achieve full automaticity and the animals were not intact. Additionally, heart edge-tracing of top-viewing videos has provided pupal heartbeat analysis, as shown by Wessels and Bodmer^25^.

OCM has then emerged as an important optical method with noninvasiveness and high spatiotemporal resolution. With this imaging method, all developmental stages can be imaged, and larvae and pupae can be returned undamaged for further studies. An algorithmic method has also been employed to analyze OCM M-Mode recordings of heart function^26^. In this method, heart segmentation is generated based on edge detection and principal component analysis. The authors used this technique to quantify heart rate but did not characterize any other parameters. By labeling the full 2D heart cross section in each OCM frame, our noninvasive technique can extract additional information, such as the heart wall velocity and heart area, enabling automatic heart function quantification based on noninvasive techniques like OCM.

Our group employs deep learning methods to segment *Drosophila* heart OCM videos^27,28^. To measure the quality of the segmentation, we use the intersection over union (IOU) between the predicted mask and the ground truth provided by human labels. The neural networks described below were all trained on a cropped region of the OCM video that contains the *Drosophila* heart and has been resized to 128 × 128 pixels. The first iteration of FlyNet, developed in 2018, consisted of a fully convolutional UNet architecture that achieved 85% testing accuracy^27^. We further developed this model to use convolutional LSTM layers to capture both spatial and temporal information^28^. FlyNet 2.0 processes whole video sequences, rather than individual frames, improving the segmentation accuracy to 92%. Since the publication of FlyNet 2.0 in 2020, our group has continued to optimize the model: the current version, called FlyNet 2.0+, achieves up to 97% accuracy on high quality videos. This optimized model uses advanced hardware to expand the network size while simultaneously decreasing the training and prediction times. Using an Nvidia RTX 3090 GPU, all 4000 OCM video frames of a beating *Drosophila* heart can be segmented in 5 seconds, fast enough for real time processing. The unique dataset we present here will enable other cardiac researchers using *Drosophila* models to increase image analysis accuracy and efficiency while reducing dependence on manual labeling and processing.

Directly, the development of FlyNet2.0+ can benefit rapid screening, disease modelling and heart disturbance monitoring in *Drosophila* cardiovascular research^29–33^. The capability of real-time processing will remove previous technical limitations and shorten the waiting interval between batches of experiments. This, along with improved accuracy, will enable new high throughput analysis that does not require human dissection of flies or manual labeling, eliminating the two most time-consuming data analysis steps, reducing human bias in segmentation. Researchers can take advantage of this low latency processing to increase sample sizes which will facilitate new experiments not possible previously.

## Methods

### Sample preparation

The dataset presented here contains videos of the beating *Drosophila* hearts we collected over the last few years across multiple experiments^4,5,20,34^. To train a generalized model that can be used for image segmentation and analysis of various physiological conditions we included imaging data of *Drosophila* at different developmental stages (larvae, pupae, and adult flies) and with different genetic backgrounds, and, therefore, with different heartbeat dynamics.

For optogenetic experiments, we obtained progeny w*; P{y+^*t7.7*^ *w*^+*mC*^ = *UAS-ReaChR}su(Hw)attP5/*+*; P{y*^+*t7.7*^ *w*^+*mC*^ = *GMR88D05-GAL4}attP2/*+ from the cross between stocks #48396 and #53748 (Bloomington Drosophila Stock Center) expressing ReaChR opsin in the heart. Progeny w*; P{y^+*t7.7*^ *w*^+*m*^*C* = *UAS-eNpHR-YFP}attP2/ P{y[* + *t7.7] w*^+*mC*^ = *GMR88D05-GAL4}attP2* from the cross between BDSC stocks #48396 and #41752 expresses NpHR opsin in *Drosophila* heart. The animals were used in the experiment to mimic cardiac arrhythmias. Here, ReaChR^35^ and NpHR^36^ are light-sensitive microbial opsins that can regulate cardiac activities during optical stimulations at specific wavelengths. ReaChR, the red-activatable channelrhodopsin, allows selective depolarization to induce cardiac contraction when expressed heart-specifically, while NpHR, the halorhodopsin from *Natronomonas*, enables neuronal inhibitions in targeted heart tissue, achieving heartbeat freezing^34^. y*w^*67c23*^ flies were cultured on regular cornmeal fly food; opsin expressing flies were reared on semi-defined fly media containing 1 mM of all-*trans* Retinal (ATR) to allow the opsins to function properly^5^.

We performed RNAi mediated ubiquitous CG3165 knock down. *w*^1118^*; P{GD*1*0318}v25784* males (v25784, Vienna Drosophila Resource Center) were crossed to *y*^*1*^ w*; P{Act5C-GAL4-w}E1/CyO (#25374, Bloomington Drosophila Stock Center) females. Progeny *P{Act5C-GAL4-w}E1/*+*; P{GD10318}v25784/*+ (adult flies) were selected for the heart functional characterization using OCM. Flies were reared on regular cornmeal fly food.

The OCM imaging quality is better at larvae and early pupae which have translucent white body cuticle compared to darker late pupae and adult flies, however the higher light absorption in late pupae and adult flies does not hinder segmentation. To mount the larval and pupal stages, the body surface was first brushed clean, then the animals were attached, dorsal side up, to double-sided tape on a glass microscope slide. Adult flies, on the other hand, were first anesthetized with CO_2_ or FlyNap and mounted, dorsal side up, on a thin layer of rubber cement on a glass slide. Then their wings were spread and stuck down to the glue, using tweezers. The mounting procedure minimized unwanted movement, and the dorsal uppermost positioning allowed unobstructed viewing.

### OCM imaging

**Fig. 1:**
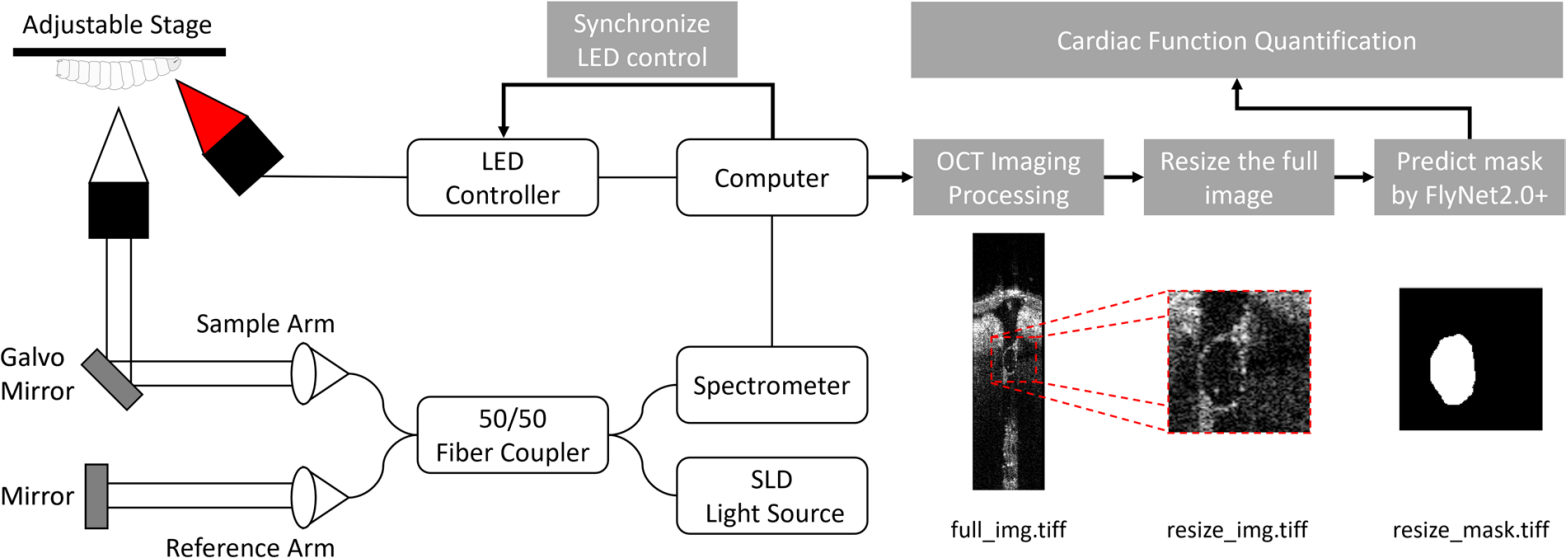
Schematic overview of the imaging and processing procedure. The left side depicts the configuration of the OCM system with a LED light illuminating the sample. The right side shows the order in which the output files are produced after the imaging is finished.

Prior to imaging, all mounted *Drosophila* specimens were placed on an adjustable sample stage, with the slide and attached fly specimen oriented longitudinally along the y-transverse direction of the OCM scanning laser, as shown in Fig. 1. For larvae and early pupae, the sample stage was adjusted to align the cross section with the A7 segment of the heart. This segment, one of the larger segments of the tubular heart in early development, has very distinguishable heart walls. As the fly gets older, the A7 segment narrows and a conical shape develops, with the A1 segment becoming the largest. Therefore, we imaged the A1 segment for late pupae and adult flies. The scanning range was set to cover ~0.3 mm in the transverse direction. In tissue with a 10x lens we can achieve an axial resolution of ~3.3 μm and a lateral resolution of ~2.3 μm. The system parameters were set to 4000 B-scans, with 128 A-scans per B-scan, and the imaging lasted for ~32 seconds. For datasets from the optogenetic pacing experiments, red (617 nm) LEDs illuminated the heart, synchronized with the OCM acquisition.

### Processing

OCM recordings of the beating fly hearts are 32 seconds in duration for each measurement. Each recording comprises 4000 frames, with each frame measuring 701 by 128 pixels. These OCM videos are stored in the stacked TIFF file format. Although this full-sized video can be used directly, cropping to closely contain just the heart region can reduce the input size to the FlyNet. No information about the heart is lost, and the reduced data size helps stay within the memory constraints of GPUs. A user can use any cropping software to create a bounding box around the heart region of the video. Data from this smaller region of interest is then extracted and interpolated to a resized 4000 × 128 × 128 video using OpenCV in Python.

Next, the resized video is fed through our FlyNet software to predict the 4000 segmentations of the heart region. Each of these segmentations is manually reviewed, and incorrectly classified pixels are corrected manually using segmentation code written in Python to remove areas outside the heart and fill in areas that are blank inside the heart. With this procedure, easily classified frames are processed automatically, and human labeling is required only for challenging scenarios. In this way, we have been able to grow our dataset and iteratively improve the model by correcting misclassified images. After the segmentation mask has been verified as accurate, a separate program converts and interpolates the mask for the resized image to a mask for the full-size image. Each sample has two mask files, a 4000 × 600 × 128 file for the full image and a 4000 × 128 × 128 file for the resized image.

With an accurate mask, we can finally extract heart parameters. Using the calibration generated for a specific OCM system, we convert pixel numbers to physical measurements. From this, we can calculate the area of one pixel in micrometers and then multiply by the number of pixels in the mask to get the heart area at one time point. When the area is plotted over time, it will fluctuate in a periodic manner, and the time points of the maximum or minimum areas can be analyzed to extract the heart rate. The maximum and minimum areas also identify the end systolic and diastolic phases. From this, fractional shortening is calculated as the percentage difference between the area at the end diastolic and systolic phases. Arrythmia index can be calculated from this as a measure of heart rhythmicity, defined as the standard deviation of the heart rate divided by the median heart rate^22^.

### Training and predicting

OCM videos were collected from 112 samples in three developmental stages (26 larvae, 39 early pupae, and 47 adults). To train the neural networks, these samples were split into 80% training, 10% validation, and 10% testing. Further validation was performed on an additional 101 samples. To fully automate the segmentation process, two different neural networks were trained on this data, using TensorFlow. The first neural network, trained on the full-size images, produces a preliminary segmentation to find the heart region of the video. This prediction is used to automatically crop and resize the video to be 128 × 128 pixels centered on the heart. The resized video is fed into the second neural network, FlyNet 2.0+, that was trained on square videos focused on the heart region. This model segments the heart accurately to reliably perform the cardiac analysis described in the Background section.

**Fig. 2:**
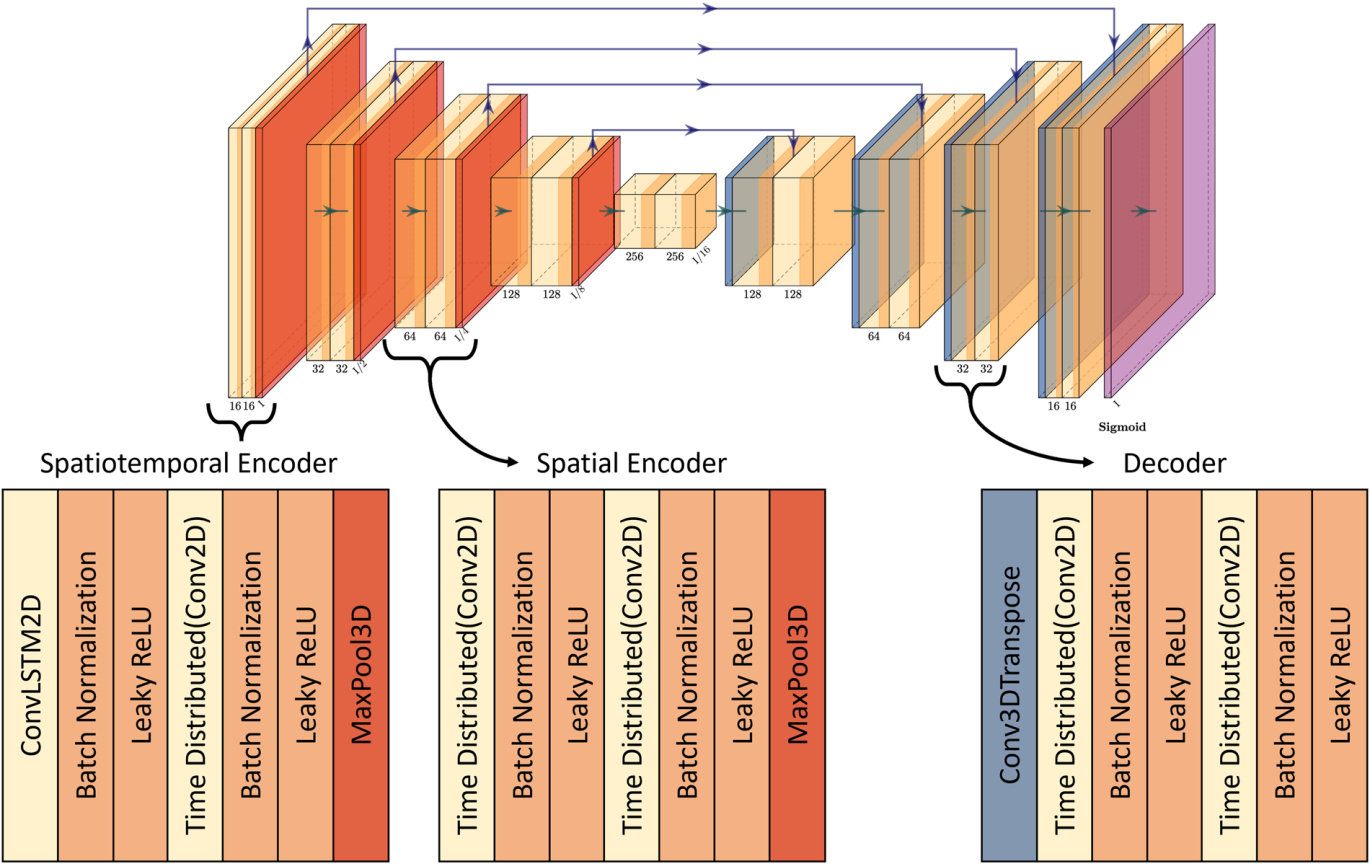
FlyNet 2.0+ model architecture, with block diagrams for the two encoders and the decoder. Each layer feeds forward to the next layer and the skip connections are shown on top.

FlyNet 2.0+ made multiple improvements to the model architecture but retains many components from past model iterations^27,28^. Additionally, the training and prediction code was upgraded to take advantage of modern hardware, reduce memory overhead, and speedup processing time. Some major changes to the programs include updating to TensorFlow 2 for newer layers and loss functions, using a generator to load the data in real time, batching the training and prediction code to achieve high utilization while staying within memory limits, and hyperparameter tuning to achieve real-time processing. The model still largely resembles the UNet architecture, with an encoder and decoder bridged together with skip connections, as shown in Fig. 2. However, the encoder block and decoder block have been improved. The encoder starts with the spatiotemporal encoder block which processes the images sequentially. The first layer of the encoder block is the LSTM 2D convolution to capture the temporal information. The remaining layers in the encoder block are wrapped in a time distributed layer, so they are applied to the frames in parallel and have no time dependency. Reducing the number of LSTM layers improves performance by eliminating sequential calculations. After the LSTM layer, batch normalization is applied before a 2D convolution. A second layer of batch normalization is applied before the leaky ReLU activation and 2D max pooling. After the first two spatiotemporal encoder blocks, there are standard spatial encoder blocks that replace the LSTM layer with a normal 2D convolution. Again, this is to limit the number of sequential calculations, only placing the LSTM layers in critical locations where temporal information is prominent. The decoder block has no LSTM layers and therefore is applied in parallel to every time slice. The decoder block starts with a transpose 2D convolution followed by concatenating the skip connections. A LSTM convolution is replaced by a normal 2D convolution, but the remainder of the decoder block is the same as the encoder block, excluding the max pooling layer.

To train FlyNet 2.0+, the Adam optimizer was used, with a learning rate of 0.001. Log-Cosh Dice Loss was chosen because it is a convex function, and because it builds on the Dice Coefficient, a very similar measurement to IOU. To allow for a variety of augmentations during training, a generator was used to load full-size files and apply a unique crop and resize to each 32-frame video sequence. Using the mask for the full-size images, we could ensure that the whole heart was within the frame for each of these different cropping parameters. Before being used to train the neural network and make predictions, each of the videos was centered to have a mean intensity of 0 and a standard deviation of 1.

### Statistical analysis

For comparisons of FlyNet 2.0 and FlyNet 2.0+, a two-tail, two-sample z-test for sample means was performed using alpha = 0.05. For CG3165 knockdown experiments, and a two-sided student’s t-test was performed. Results were deemed significant when p < 0.05.

## Data Records

**Fig. 3:**
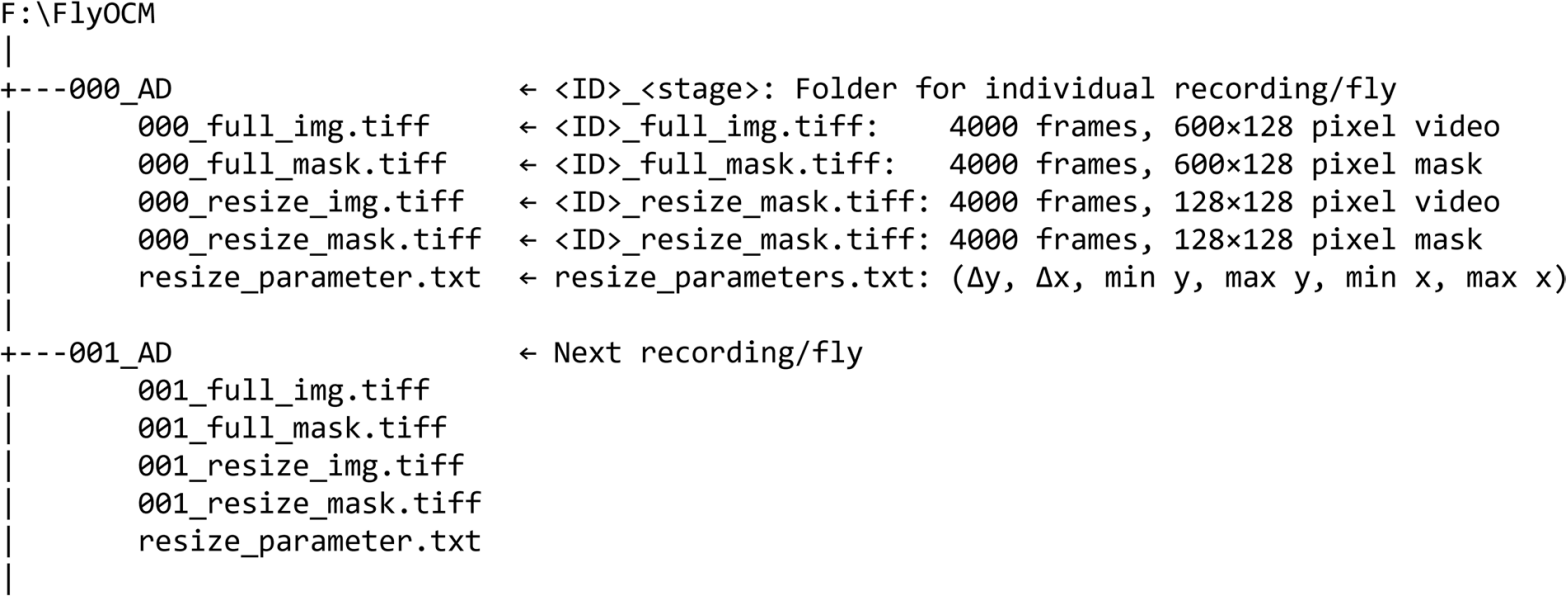
File structure for fly dataset. Each individual dataset is contained in a single folder with 5 files.

Our dataset is publicly available at *figshare* and can be downloaded as a zip file^37^. The file structure of the dataset is shown in Fig. 3. After the dataset is unzipped, each of the samples will have a unique ID and its own folder. The folder name is <ID>_<age>, where LA is larva, EP is early pupa, and AD is adult. All the videos and masks are saved in 4000 frame TIFF files, except for adults which are only 2000 frames. Table 1 shows the breakdown of the complete dataset by age and number of fames. <ID>_full_img.tiff is the original 600 × 128 pixels image, and <ID>_full_mask.tiff is the segmentation of the heart in this image. Each of the pixels in the mask has a value of either 0, corresponding to background, or 255, corresponding to the heart region. Similarly, <ID>_resize_img.tiff contains a 128 × 128 resized video, and <ID>_resize_mask.tiff is the corresponding mask. Cropping information is stored in each folder under the name resize_parameters.txt. The text file contains 6 numbers corresponding to delta y, delta x, min y, max y, min x, max x where the min and max define the starting and ending pixel on the full-size image.

**Table 1:** Fly heart OCM videos for different developmental stages.

	Videos	Frames
Larva	57	192,000
Early Pupa	52	204,000
Adult	104	208,000
**Total**	**213**	**604,000**

## Technical Validation

### Ground truth validation

**Fig. 4:**
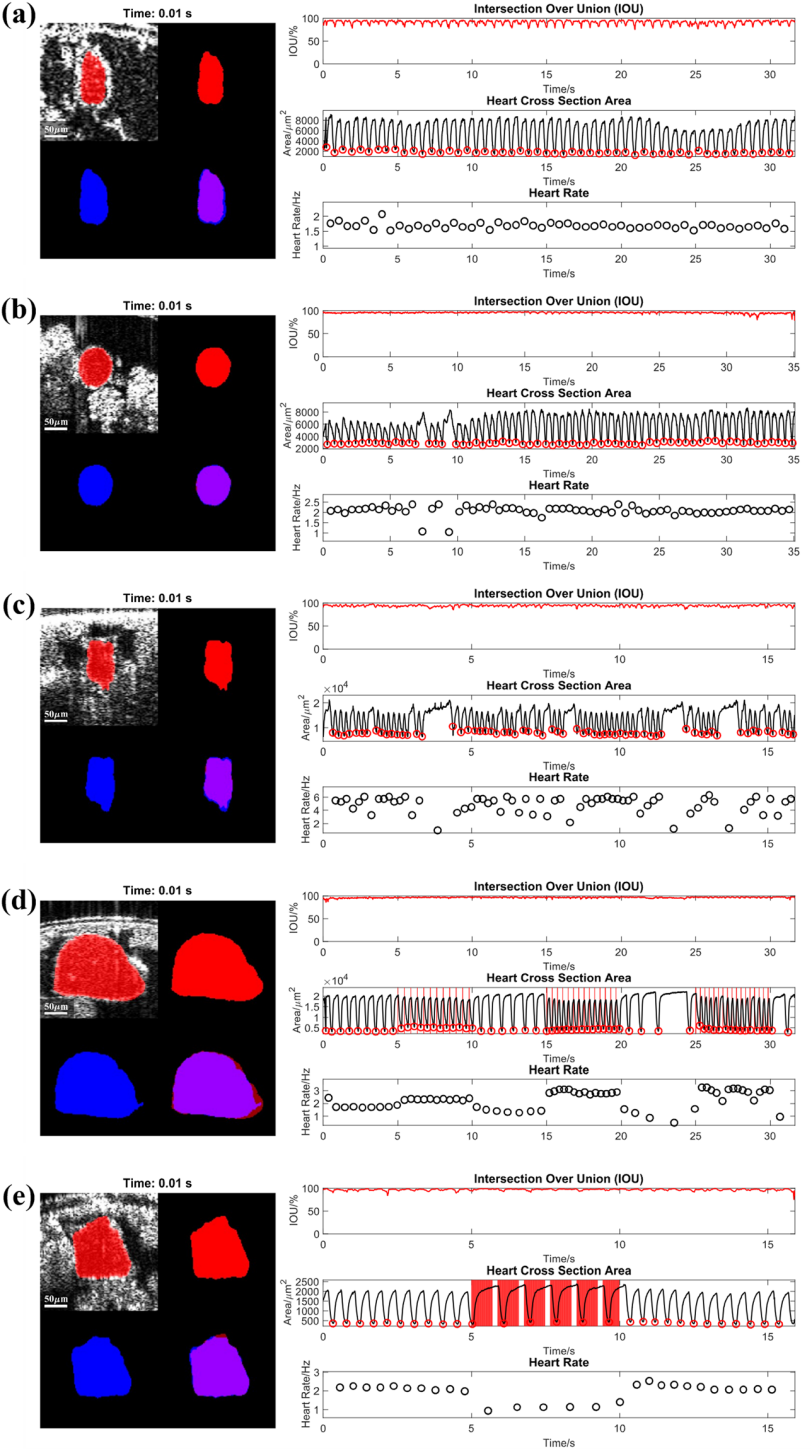
Comparison of the ground truth mask (red) and the FlyNet 2.0+ mask (blue). Each of the five panels shows a preview of Supplementary File 1–5. Within each panel, the top left quadrant shows the ground truth overlayed on the resized image, while the bottom right shows the two masks overlayed on each other to highlight differences in the segmentation. On the right is the IOU, the area of heart, and the heart rate for each frame plotted over time. (**a**–**c**) show a larva, an early pupa and an adult fly respectively with no light stimulation. (**d,e**) show optogenetics pacing experiments on an early pupa with an excitatory opsin, ReaChR and an inhibitory opsin, NpHR, respectively. The vertical red lines correspond to when the light pulses were executed. The heart rate increased or decreased to the designed frequency respectively.

To assess the quality of the labeling procedure, a randomly selected set of images was labeled by an expert with no prior predicted mask. Comparing these labels to the traditional procedure of correcting predicted masks allowed us to analyze the model’s influence on label quality. Additionally, these images were labeled on the full-size files, with no interpolation to validate the cropping and resizing procedure. Comparing the expert-labeled masks to the model-corrected masks yielded an IOU score of 88%, demonstrating that the labeling procedure accurately segments the heart. The minute differences in the masks can be attributed to poorly defined boundaries as well as small deviations introduced by the interpolation. As seen in Fig. 4 (videos are available as supplemental materials), the small differences in the mask have little effect on the measured area and therefore the cardiac parameters are still accurate despite these discrepancies. The IOU appears periodic, but this is due to the nature of IOU calculation, where pixel shifts in smaller areas will have a greater impact on the resultant score. Because human labelers need to manually segment the heart in each frame, it is nearly impossible to get an IOU of over 90% because humans are not capable of pixel level precision.

### Segmentation quality analysis

Additionally, the quality of the segmentations produced by FlyNet 2.0+ further validates the label quality. FlyNet 2.0+ can achieve an average IOU of around 92% and as high as 96%, which is within the same range of variability seen between any two human labelers. In other words, the expert annotator mask, the predicted and corrected mask, and the FlyNet 2.0+ mask are labeling the same heart region. The differences between these masks, small deviations in the boundary of the heart wall, are not significant enough to impact cardiac analysis. Figure 4 demonstrates that FlyNet is able to segment the fly heart across different conditions, such as various developmental stages (Fig. 4a–c), and with enhanced or suppressed heart function manipulation using optogenetic methods (Fig. 4d,e). All subfigures correspond to the supplementary videos (Supplementary Video 1–5) that show the dynamic changes of heart rate and IOU fluctuation. We can conclude that the procedure of resizing OCM videos, predicting masks, and correcting masks produces a high-quality ground truth that can be used to train an accurate segmentation algorithm.

**Fig. 5:**
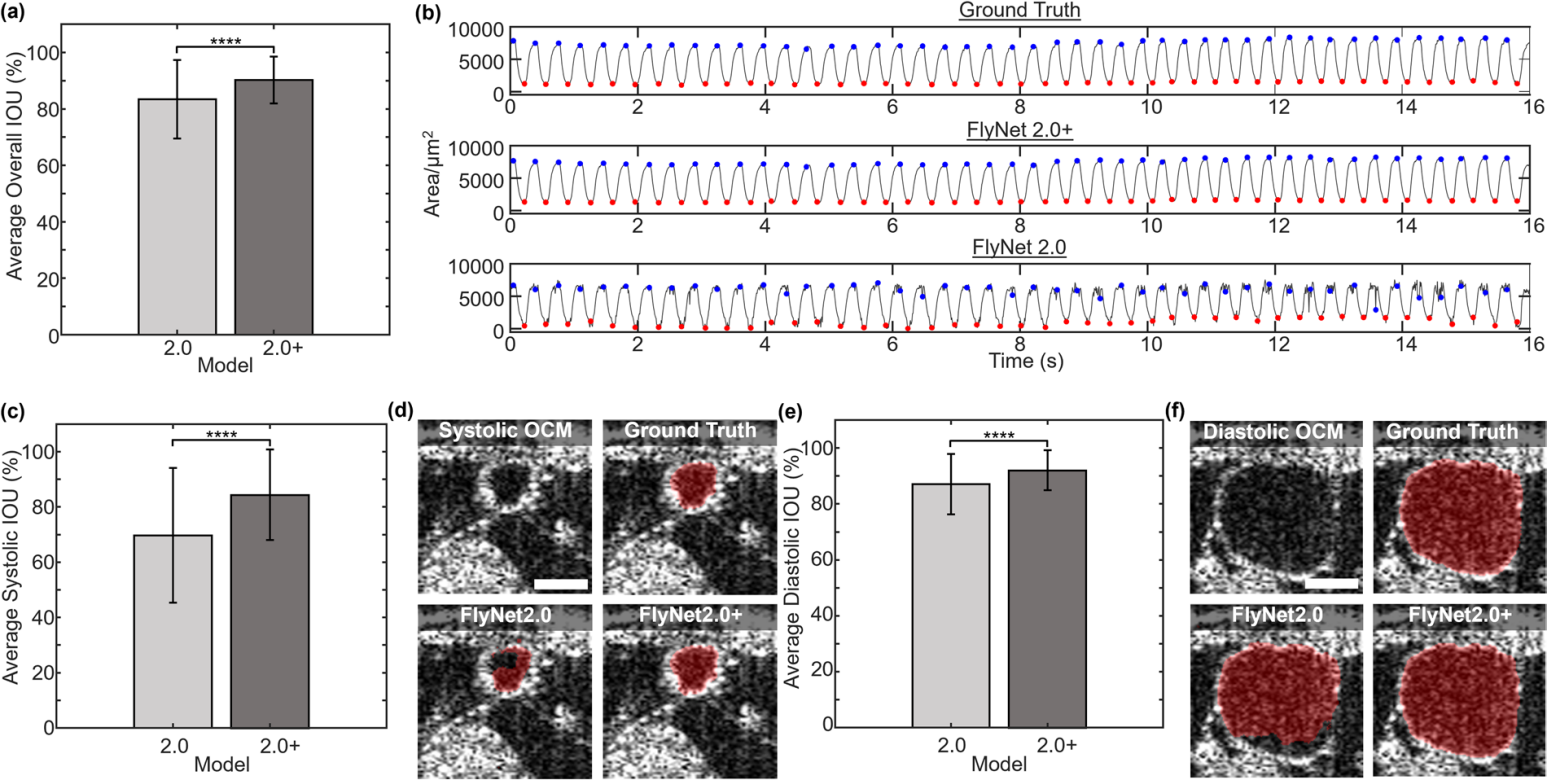
Performance Comparison of FlyNet 2.0+ with previous version FlyNet 2.0. (**a**) A bar plot comparing the overall average IOU of FlyNet 2.0 and FlyNet 2.0+. The error bars indicate the range of standard deviation. (**b**) Area plotted over time, where the correct systolic indices are labeled with red dots and correct diastolic indices labeled with blue dots. (**c**) A bar plot comparing the end systolic IOU between the two models. The error bars indicate the range of standard deviation. (**d**) Differences in the end systolic state between the ground truth, FlyNet2.0, and FlyNet2.0+. The scale bar for each image is shown on the raw OCT image as 50 µm. (**e**) Comparison of the end diastolic IOU, with error bars indicating standard deviation, and (**f**) the differences in the end diastolic state between ground truth and the two models. Scale bar 50 µm. ****p < 0.0001.

For additional comparison, the models were compared in a study of n = 217 image sets with high quality ground truth segmentations. Each image set was predicted on FlyNet 2.0 and FlyNet 2.0+, and the time/frame, overall IOU, and IOU at end systolic and end diastolic points was noted. The IOU comparison in Fig. 5a shows that overall, the IOU improves from FlyNet 2.0 significantly (p < 0.0001), from an average of about 83% to an average of about 90%. This improvement in segmentation quality will greatly reduce time spent correcting masks by hand. Figure 5b shows a time plot of an example recording of the ground truth mask, FlyNet2.0+ mask, and FlyNet2.0 mask. Both the ground truth and FlyNet2.0+ areas show good agreement with the area at the correct systolic and diastolic indices, but the FlyNet2.0 area plot introduces a level of error.

End systolic (Fig. 5c,d) and end diastolic (Fig. 5e,f) IOUs were also calculated to show the improvement breakdown at the end of each phase. The end systolic IOU significantly (p < 0.0001) improved from an average of 70% in FlyNet 2.0 to 84% in FlyNet 2.0+. End systolic IOU has smaller area by definition, so an improvement in prediction accuracy in this case will drastically improve the IOU. The end diastolic IOU also significantly (p < 0.0001) improved from 87% in FlyNet 2.0 to 92% in FlyNet 2.0+.

### Performance on different beating patterns

**Fig. 6:**
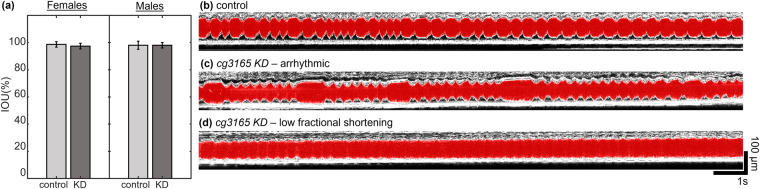
Segmentation performance on abnormal cardiac function. (**a**) Comparison of the mean IOU from *yw* and *cg3165* KD flies, separated as male and female. (**b**) An M-mode image of a healthy *yw* 1 week old fly, with the segmented heart region shown in red. (**c**) An M-mode of a 1-week-old fly with *cg3165* KD, whose heart function has low fractional shortening. The FlyNet 2.0+ segmentation of the heart region is in red. (**d**) An M-mode of a 1-week-old fly with *cg3165* KD, whose heart function is arrhythmic. The FlyNet 2.0+ segmentation of the heart region is in red.

To demonstrate the robustness of our model on a dataset that is outside normal cardiac function, we performed analysis of a set of data collected from adult flies where CG3165 protein was ubiquitously depleted by RNAi. OCM monitored heart function and FlyNet 2.0+ provided rapid segmentation for further analysis. We imaged the heart function of control and *cg3165* RNAi 1 week old adult flies, separated as male and female. Figure 6a shows that FlyNet 2.0+ still performs with the same level of accuracy as with control flies of the same age. Figure 6b–d demonstrate the ability of FlyNet 2.0+ to accurately identify abnormal heart function, including cases of arrythmia (Fig. 6c) and lower fractional shortening (Fig. 6d). Thus, FlyNet2.0+ can perform accurately on a wide range of beating and contraction patterns.

## Usage Notes

While this automated labeling technique is powerful, it has some limitations. First, the accuracy of the labels is dependent on high quality OCM recordings. OCM can be prone to visual artifacts and obstructions that can hurt the visibility of the heart. In all developmental stages, the largest area of the heart is used for recording, so the model may not perform as accurately in different regions. Additionally, in the larval stage, movement may occur which obscures the heart region. If the heart is obscured in the recording it can be impossible to produce accurate ground truth labels, making the neural network perform poorly in these situations. We suggest taking multiple repeats of each dataset and fine tuning the recording location to avoid these artifacts.

To open and view the TIFF files we recommend using ImageJ/Fiji (https://imagej.net/software/fiji/) which is an open source and free platform for viewing medical images. The images are saved without any color channels, making both the images and masks grayscale. To overlay the mask on the image, it must first be converted to a color image and then the mask can be added to the red channel. Additionally, saved pixel values are cast to be between 0 and 255 for optimal viewing. Upon loading the images into the Python processing code, masks are converted to be 0 or 1, and the videos are centered at 0 with a standard deviation of 1.

Supplementary videos can be found on figshare^37^.

## Data Availability

All the models were trained using Python 3.9 and Tensorflow 2.10. Models were trained locally on a 3090 GPU with 24 GB of memory. A GitHub repository which contains the Jupyter notebook for training is available at https://github.com/mattfishman/Drosophila-Heart-OCM. To run the code, first download the data and clone the repository. There is a requirements.txt file within the repository that contains all the dependencies. Next, use the utilities to generate a pickle file by changing the path inside create_pickle.py to point to the parent directory that contains all the flies. Insert the pickle file path into the training notebook and run all cells begin training. Our trained model has been provided as a starting place for training, but users may opt to train without this initialization. This notebook will output the model as a.h5 file. The path to the trained model can then be inserted into processing_utils.py to use this model to predict the segmentation on new images. Additional details can be found in the README file within the repository for how to run additional code to produce cardiac parameters.

## References

[1] Bier E, Bodmer R (2004). Drosophila, an emerging model for cardiac disease. Gene.

[2] Reiter LT, Potocki L, Chien S, Gribskov M, Bier E (2001). A Systematic Analysis of Human Disease-Associated Gene Sequences In Drosophila melanogaster. Genome Res..

[3] Hayashi S, Kondo T (2018). Development and Function of the Drosophila Tracheal System. Genetics.

[4] Men, J. *et al*. Drosophila Preparation and Longitudinal Imaging of Heart Function *In Vivo* Using Optical Coherence Microscopy (OCM). *JoVE J. Vis. Exp*. e55002, 10.3791/55002 (2016). 10.3791/55002 PMC5226401 28060288

[5] Gracheva, E. *et al*. Developing Drosophila melanogaster Models for Imaging and Optogenetic Control of Cardiac Function. *JoVE J. Vis. Exp*. e63939, 10.3791/63939 (2022). 10.3791/63939 PMC9825051 36094265

[6] Thomas D, Duguid G (2004). Optical coherence tomography—a review of the principles and contemporary uses in retinal investigation. Eye.

[7] Everett M, Magazzeni S, Schmoll T, Kempe M (2021). Optical coherence tomography: From technology to applications in ophthalmology. *Transl*. Biophotonics.

[8] Hendon CP, Lye TH, Yao X, Gan Y, Marboe CC (2019). Optical coherence tomography imaging of cardiac substrates. Quant. Imaging Med. Surg..

[9] Araki M (2022). Optical coherence tomography in coronary atherosclerosis assessment and intervention. Nat. Rev. Cardiol..

[10] Oosterveer TTM, van der Meer SM, Scherptong RWC, Jukema JW (2020). Optical Coherence Tomography: Current Applications for the Assessment of Coronary Artery Disease and Guidance of Percutaneous Coronary Interventions. Cardiol. Ther..

[11] Gora MJ, Suter MJ, Tearney GJ, Li X (2017). Endoscopic optical coherence tomography: technologies and clinical applications [Invited]. Biomed. Opt. Express.

[12] Kim J (2020). Flexible endoscopic micro-optical coherence tomography for three-dimensional imaging of the arterial microstructure. Sci. Rep..

[13] Tang Y, Anandasabapathy S, Richards-Kortum R (2021). Advances in optical gastrointestinal endoscopy: a technical review. Mol. Oncol..

[14] Welzel J (2001). Optical coherence tomography in dermatology: a review. Skin Res. Technol. Off. J. Int. Soc. Bioeng. Skin ISBS Int. Soc. Digit. Imaging Skin ISDIS Int. Soc. Skin Imaging ISSI.

[15] Olsen J, Holmes J, Jemec GB (2018). Advances in optical coherence tomography in dermatology-a review. J. Biomed. Opt..

[16] Ulrich M (2016). Dynamic Optical Coherence Tomography in Dermatology. Dermatology.

[17] Hsieh Y-S (2013). Dental Optical Coherence Tomography. Sensors.

[18] Shimada Y, Yoshiyama M, Tagami J, Sumi Y (2020). Evaluation of dental caries, tooth crack, and age-related changes in tooth structure using optical coherence tomography. Jpn. Dent. Sci. Rev..

[19] Machoy M (2017). The Use of Optical Coherence Tomography in Dental Diagnostics: A State-of-the-Art Review. J. Healthc. Eng..

[20] Alex A, Li A, Tanzi RE, Zhou C (2015). Optogenetic pacing in Drosophila melanogaster. Sci. Adv..

[21] Ocorr K, Vogler G, Bodmer R (2014). Methods to assess Drosophila heart development, function and aging. Methods San Diego Calif.

[22] Fink M (2009). A new method for detection and quantification of heartbeat parameters in Drosophila, zebrafish, and embryonic mouse hearts. BioTechniques.

[23] Paternostro G (2001). Age-Associated Cardiac Dysfunction in Drosophila melanogaster. Circ. Res..

[24] Mönck H (2017). A new method to characterize function of the Drosophila heart by means of optical flow. J. Exp. Biol..

[25] Wessells, R. J. & Bodmer, R. Screening assays for heart function mutants in Drosophila. *BioTechniques* **37**, 58–60, 62, 64 passim (2004). 10.2144/04371ST01 15283201

[26] Lee C-Y, Wang H-J, Jhang J-D, Cho I-C (2019). Automated drosophila heartbeat counting based on image segmentation technique on optical coherence tomography. Sci. Rep..

[27] Duan L (2018). Segmentation of Drosophila heart in optical coherence microscopy images using convolutional neural networks. J. Biophotonics.

[28] Dong Z (2020). FlyNet 2.0: drosophila heart 3D (2D + time) segmentation in optical coherence microscopy images using a convolutional long short-term memory neural network. Biomed. Opt. Express.

[29] Holmes J (2009). *In vivo* real-time optical coherence tomography imaging of Drosophila for cardiovascular research. Nat. Methods.

[30] Wolf MJ (2006). Drosophila as a model for the identification of genes causing adult human heart disease. Proc. Natl. Acad. Sci..

[31] Choma MA, Izatt SD, Wessells RJ, Bodmer R, Izatt JA (2006). *In Vivo* Imaging of the Adult Drosophila melanogaster Heart With Real-Time Optical Coherence Tomography. Circulation.

[32] Tsai, M.-T. *et al*. Dynamic Monitoring of the Heart Beating Behaviors of Drosophila with Optical Coherence Tomography. in *Conference on Lasers and Electro-Optics 2010 (2010), paper JWA81* JWA81. 10.1364/CLEO.2010.JWA81 (Optica Publishing Group, 2010).

[33] Mt, T. *et al*. Noninvasive imaging of heart chamber in Drosophila with dual-beam optical coherence tomography. *J. Biophotonics* **6** (2013). 10.1002/jbio.201200164 23192969

[34] Men J (2020). Non-invasive red-light optogenetic control of Drosophila cardiac function. Commun. Biol..

[35] Lin JY, Knutsen PM, Muller A, Kleinfeld D, Tsien RY (2013). ReaChR: A red-shifted variant of channelrhodopsin enables deep transcranial optogenetic excitation. Nat. Neurosci..

[36] Gradinaru V, Thompson KR, Deisseroth K (2008). eNpHR: a Natronomonas halorhodopsin enhanced for optogenetic applications. Brain Cell Biol..

[37] Fishman M (2023). figshare.

